# Getting closer to the coronary arteries: A bibliometric analysis of CT-based adipose tissue imaging in coronary artery disease

**DOI:** 10.1097/MD.0000000000040592

**Published:** 2024-11-22

**Authors:** Jiayi Fu, Rajiv Baichoo, Xing Xiong, Wenyue Shen, Kai Jin, Xiaojun Guan, Qijing Zhou, Xiaojun Xu

**Affiliations:** a Department of Radiology, The Fourth Affiliated Hospital of School of Medicine, and International School of Medicine, International Institutes of Medicine, Zhejiang University, Yiwu, China; b Department of Radiology, The Second Affiliated Hospital, Zhejiang University School of Medicine, Zhejiang, Hangzhou, China; c Eye Center, The Second Affiliated Hospital School of Medicine, Zhejiang University, Zhejiang Provincial Key Laboratory of Ophthalmology, Zhejiang Provincial Clinical Research Center for Eye Diseases, Zhejiang Provincial Engineering Institute on Eye Diseases, Hangzhou, China.

**Keywords:** adipose tissue, bibliometrics, computed tomography, coronary artery disease

## Abstract

The aim of this study is to conduct a comprehensive bibliometric analysis of CT-based adipose tissue imaging related to coronary artery disease (CAD) to investigate the dynamic development of this field. Web of Science Core Collection was used as our data source to identify relevant documents limited to articles or review articles and written in English with no time restrictions. Then we analyzed the whole trend of publications and utilized VOSviewer and Bibliometrix to conduct a bibliometric analysis including citations, keywords, countries, institutions, authors as well as co-citation analyses of cited references and sources. A total of 629 documents including 560 articles and 69 reviews from 1992 to 2023 were included. The trend of publications was divided into 3 phases and overall exhibited a constant rise. Based on the co-occurrence network of keywords analysis, 3 clusters centered on visceral, epicardial, pericoronary adipose tissue respectively and 1 cluster related to cardiovascular risk factors were identified, meanwhile determining the evolution of fat research. Co-citation analysis suggested that sources were divided into metabolism-related and cardiovascular-related journals. The USA ranked first with 228 documents and 12,086 citations among 47 countries and 1002 institutions, both at the author and institutional levels. In conclusion, this study demonstrated the thriving research field of the impact of CT-based adipose tissue assessment on coronary artery disease, offering a better understanding of the current state of research and valuable insights for future studies and collaborations.

## 1. Introduction

Adipose tissue regulates cardiovascular health due to its active participation in metabolic and inflammatory processes through secreting various bioactive molecules in endocrine and paracrine ways.^[1,2]^ In conditions such as atherosclerotic disease, pro-inflammatory signals derived from diseased vessels can directly affect adipose tissue biology and modify their phenotype.^[3]^ This bidirectional interplay between adipose tissue and the cardiovascular system lays the foundation for adipose tissue imaging as a method for assessing coronary artery disease (CAD). As a noninvasive imaging modality, coronary computed tomography angiography (CCTA) has been a first-line tool for the effective assessment of CAD, enabling visualization and assessment of adipose tissue throughout the body. The milestone study by Antonopoulos et al demonstrated the impaired adipocyte differentiation and intracellular lipid formation in pericoronary adipose tissue (PCAT) induced by vascular inflammation could be monitored using CCTA,^[2]^ which significantly opened a new chapter on cardiac adipose tissue research, and triggered a surge in CT-based adipose tissue imaging research in recent years. So far, CT-based adipose tissue imaging has provided valuable evidence for disease prediction, risk stratification and prognosis prediction of CAD, holding promise as a more powerful indicator for early management beyond traditional factors.

However, although the current research on adipose tissue is flourishing, and a large number of studies have confirmed the important value of CT-based adipose tissue imaging in CAD, few studies to date have well investigated its current research status, including the research trend, subsequent focus and future direction. Therefore, it is a suitable time to conduct a comprehensive analysis and evaluation of adipose tissue as a research hot spot to gain insights into the research landscape, identify research trends, assess research impacts and inform decision-making in academia.

Bibliometric analysis is recognized as a rigorous methodology for exploring vast quantities of scientific data.^[4]^ It allows for the objective and quantitative assessment of scholarly activities and the identifications of research trends and hot spots. After acquiring data from scientific databases such as Web of Science, as an easily accessible tool, VOSviewer and Bibliometrix can display bibliometric maps in a comprehensible manner.^[5,6]^ The purpose of this work is to perform a bibliometric analysis to present the research trends, emphasis, potential and challenges to offer a systematic overview of researches regarding CT-based adipose tissue assessment on CAD.

## 2. Materials and methods

### 2.1. Data source and search criteria

We employed Web of Science Core Collection (WoSCC) as our data source. The search was performed on December 12, 2023 and the WosCC database was updated on December 9, 2023. In order to accurately retrieve interested publications, the following search formula was applied: Topic = (adipose or fat) AND (CCTA or CTCA or CT or “coronary angiography” or “coronary computed tomography angiography” or “computed tomography”) AND (“coronary artery disease” or “coronary heart disease” or CAD or atherosclerosis or “myocardial infarction” or “coronary syndrome” or ACS or MI or “Ischemic Heart Disease” or IHD or angina).

### 2.2. Data processing

**Figure 1.**
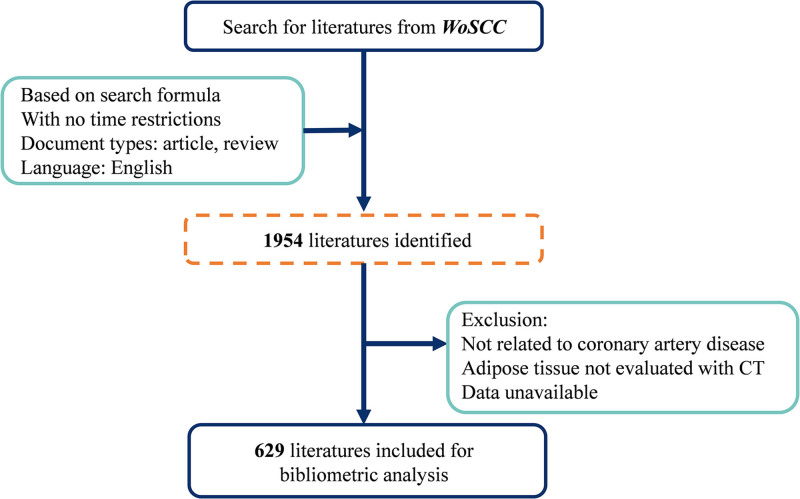
Flow chart of research data collection. WoSCC = Web of Science Core Collection.

According to the document type classified in WosCC, we kept our scope of documents limited to articles or review articles written in English with no time restrictions. To ensure the accuracy of data, we further screened the documents manually according to the title, abstract, keywords, and the full text when necessary, using the following inclusion criteria: (1) studies focused on CAD; (2) studies were involved in adipose tissue assessed by CT; (3) studies investigated the association between adipose tissue and CAD. The whole screening process is shown in Figure 1. Then we exported the full records and cited references of all documents included in this study from WoSCC in plain text form. R code (R version 4.4.1) with Bibliometrix package and VOSviewer was used for data analysis.

### 2.3. Data analysis and visualization

Based on the WoSCC database, VOSviewer, and Bibliometrix, we obtained fundamental information regarding the number of publications per year, authors, institutions, countries, keywords, publication sources, cited references, and their citations. Data aggregation was conducted in Microsoft Excel. Along with annual publications and change rate, timespan of the occurrence of keywords related to adipose tissue was constructed to determine the research trend. Hotspots are defined as keywords related to adipose tissue that showed the fastest cumulative frequency increase in the year with the highest annual publication change rate, reflecting areas of significant research interest in the field. The h-index is a metric used to measure the impact and productivity of a researcher’s scholarly work, which means a researcher have published at least h papers, and each paper has been cited at least h times.^[7]^ Scimago Graphica was applied to show the global distributions of publications and citations.

We constructed the co-occurrence network map and time overlay visualization map of keywords to identify the current hotspots and focal points in a research field. The clustering algorithm used in VOSviewer is based on the co-occurrence of keywords in the literature. By analyzing the co-occurrence patterns of keywords in the documents, the algorithm can determine the similarity between keywords and group those with similar co-occurrence patterns together to form clusters. The recognition of frequently appeared keyword combinations provides insights into the current research trends and cutting-edge areas. Moreover, the collaboration network map of the co-authorship by countries was built.

Co-citation analysis provides a method for investigating the structure and dynamic development of a field, identifying the influential articles and exploring their interconnections. Briefly, if 2 papers (or more papers) are cited simultaneously by 1 or more later papers, these 2 papers constitute a co-citation relationship. Co-citation network maps of the cited references and their journals were constructed in this study.

## 3. Results

### 3.1. Trends analysis of publications and citations

**Figure 2.**
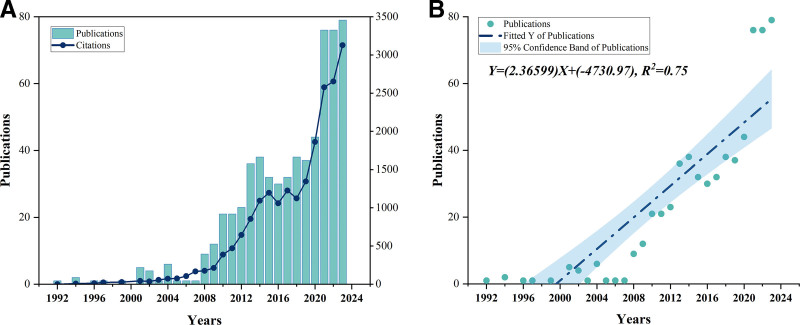
Trend analysis of annual publications in the field assessing the impact of adipose tissue on coronary artery disease using computed tomography. (A) Annual trends of publications and citations. (B) The fitting curve of publications.

**Figure 3.**
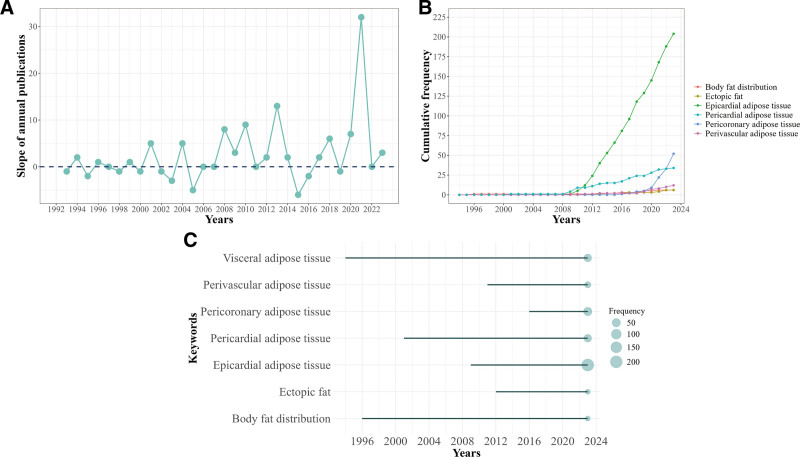
(A) The slope of annual publications. (B) The cumulative frequency of keywords related to adipose tissue over the years. (C) The timespan of the occurrence of keywords related to adipose tissue.

A total number of 629 articles published from 1992 to 2023 were identified in this study, including 560 articles and 69 reviews. As shown in Figure 2A, the plot revealed that the annual trends of publications and citations experienced a consistent upward rise and the trend of publications can be divided into 3 phases. The first phase was from 1992 to 2007, the number of annual publications was <10. From 2008 to 2016, the number of publications clearly increased. After a decline in 2015 to 2016, the number of publications returned to the above upward trajectory in 2017, indicating the beginning of the third phase (2017–2023). Based on the analysis of keywords frequency related to adipose tissue over time (Fig. 3B and C), studies from the first stage put focus on the body fat distribution and visceral adipose tissue (VAT). In the subsequent 2 phases, the research focus shifted primarily to epicardial adipose tissue (EAT) and PCAT, which respectively represented most of pericardial (PAT) and perivascular adipose tissue due to the overlap in the early use of these terms. Although its occurrence is not frequent, the emergence of the concept of ectopic fat indicates that research on adipose tissue related to CAD is gaining attention. According to the publication fitting curve (Fig. 2B), the number of publications per year generally shows a continuous growth trend. In recent years, there has been a notable acceleration in growth from 2021 to 2023 accounting for 36.7% of the total. Notably, 2021 was the year with the highest annual publications change rate (Fig. 3A), and the keywords related to adipose tissue that showed the fastest cumulative frequency increase were EAT and PCAT (Fig. 3B), becoming the hotspots in this field.

We listed top 10 most cited articles (Table 1), of which there was 1 review and 9 articles. A review written by Neeland et al in 2019 stands out with 544 citations.

**Table 1:** Top 10 most cited articles.

Title	Year	First author	Citations	Journal (document type)
Visceral and ectopic fat, atherosclerosis, and cardiometabolic disease: a position statement^[8]^	2019	Ian J. Neeland	544	Lancet Diabetes and Endocrinology (review)
Detecting human coronary inflammation by imaging perivascular fat^[2]^	2017	Alexios S. Antonopoulos	491	Science Translational Medicine (article)
Noninvasive detection of coronary inflammation using computed tomography and prediction of residual cardiovascular risk (the CRISP CT study): a post hoc analysis of prospective outcome^[9]^	2018	Evangelos K. Oikonomou	490	Lancet (article)
Association of pericardial fat, intrathoracic fat, and visceral abdominal fat with cardiovascular disease burden: the Framingham Heart Study^[10]^	2009	Amir A. Mahabadi	471	European Heart Journal (article)
Body fat distribution and insulin resistance in healthy Asian Indians and Caucasians^[11]^	2001	A. Raji	375	Journal of Clinical Endocrinology and Metabolism (article)
The association of pericardial fat with incident coronary heart disease: the Multi-Ethnic Study of Atherosclerosis (MESA)^[12]^	2009	Jingzhong Ding	356	American Journal of Clinical Nutrition (article)
Association of epicardial fat with cardiovascular risk factors and incident myocardial infarction in the general population: the Heinz Nixdorf Recall Study^[13]^	2013	Amir A. Mahabadi	346	Journal of The American College of Cardiology (article)
Contribution of visceral fat accumulation to the development of coronary artery disease in nonobese men^[14]^	1994	T. Nakamura	337	Atherosclerosis (article)
Epicardial adipose tissue and coronary artery plaque characteristics^[15]^	2010	Nikolaos Alexopoulos	246	Atherosclerosis (article)
Pericardial fat accumulation in men as a risk factor for coronary artery disease^[16]^	2001	R. Taguchi	236	Atherosclerosis (article)

### 3.2. Keywords analysis

**Figure 4.**
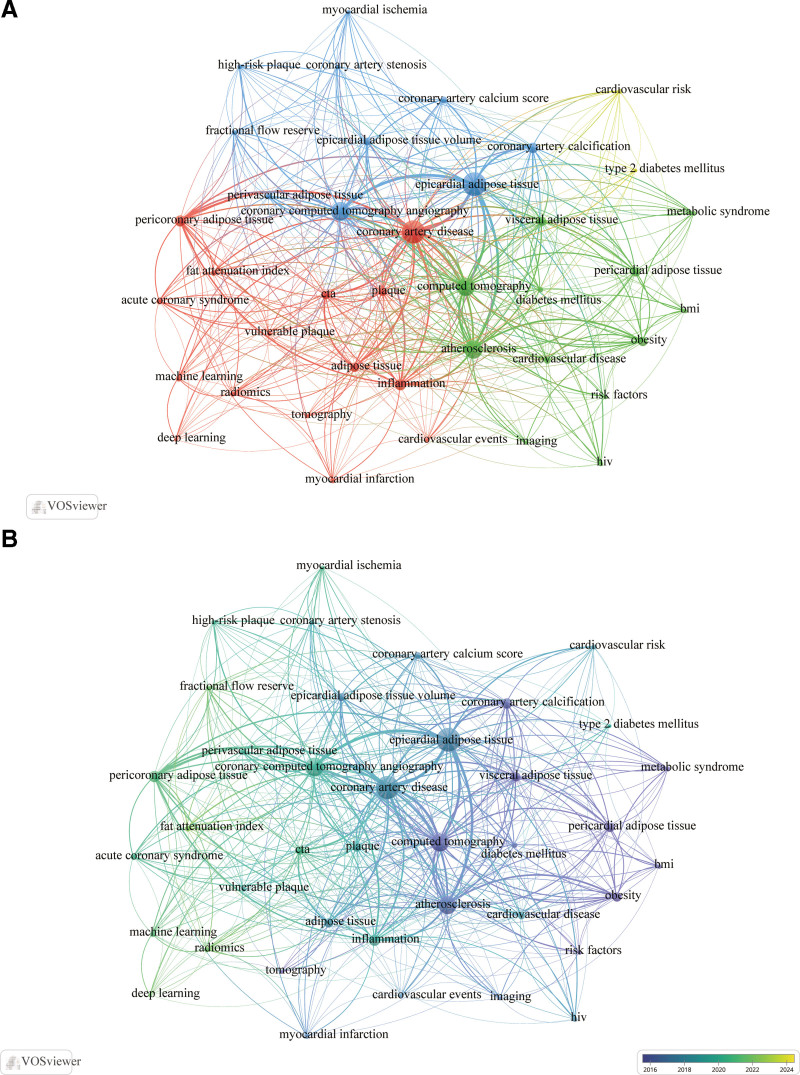
Co-occurrence network map (A) and time overlay visualization map (B) of keywords with at least 10 occurrences. The size of the nodes indicates the frequency of occurrence. The thickness of the lines represents the total number of co-occurrences with other keywords. The colors of the nodes ranging from blue to cyan indicate the sequential order of the appearance of keywords mainly from 2016 to 2023.

We extracted 39 keywords that appeared more than 10 times from all the keywords and constructed a co-occurrence network (Fig. 4a). Among these keywords, those directly related to fat included “epicardial adipose tissue” (182), “pericoronary adipose tissue” (55), “visceral adipose tissue” (41), “pericardial adipose tissue” (41) “perivascular adipose tissue” (PVAT) (23), and “fat attenuation index” (20). In Figure 4A, we can see 3 clusters shown in green, blue and red, centered on VAT/PAT, EAT, and PCAT/PVAT, respectively, and another cluster shown in yellow is related to cardiovascular risk. The results of the time overlap visualization analysis (Fig. 4B) show that VAT/PAT appeared earlier among these words, followed by EAT, and finally PCAT/PVAT.

The content of the green cluster in Figure 4A dominated the early research topics, with keywords such as “atherosclerosis,” “computed tomography,” “obesity,” “pericardial adipose tissue” and “visceral adipose tissue” having a large weight. In addition, “metabolic syndrome,” “HIV,” and “diabetes mellitus” in the green cluster are also related to CAD. In particular, “type 2 diabetes mellitus” occurred frequently together with “cardiovascular risk” shown in the yellow cluster. EAT, shown in blue, was the most frequent keyword of all and plays an active role in the study of CAD and fat. In the blue cluster, keywords related to CAD hemodynamics such as “coronary artery stenosis,” “fractional flow reserve” and “myocardial ischemia” are all related to “epicardial adipose tissue volume.” The same is true for “coronary artery calcium score” and “high-risk plaque.” In recent years, “pericoronary adipose tissue” and “fat attenuation index” in the red cluster have become hot topics as noninvasive imaging biomarkers for detecting “inflammation,” which is imperceptible with the naked eye. Keywords such as “inflammation,” “plaque,” “vulnerable plaque,” and “acute coronary syndrome” appeared frequently in the literature. In addition, “radiomics,” “machine-learning,” and “deep learning” techniques have gradually been applied to PCAT analysis, with increasing applications in risk prediction and stratification of CAD patients.

### 3.3. Co-citation analysis of cited references and sources

**Figure 5.**
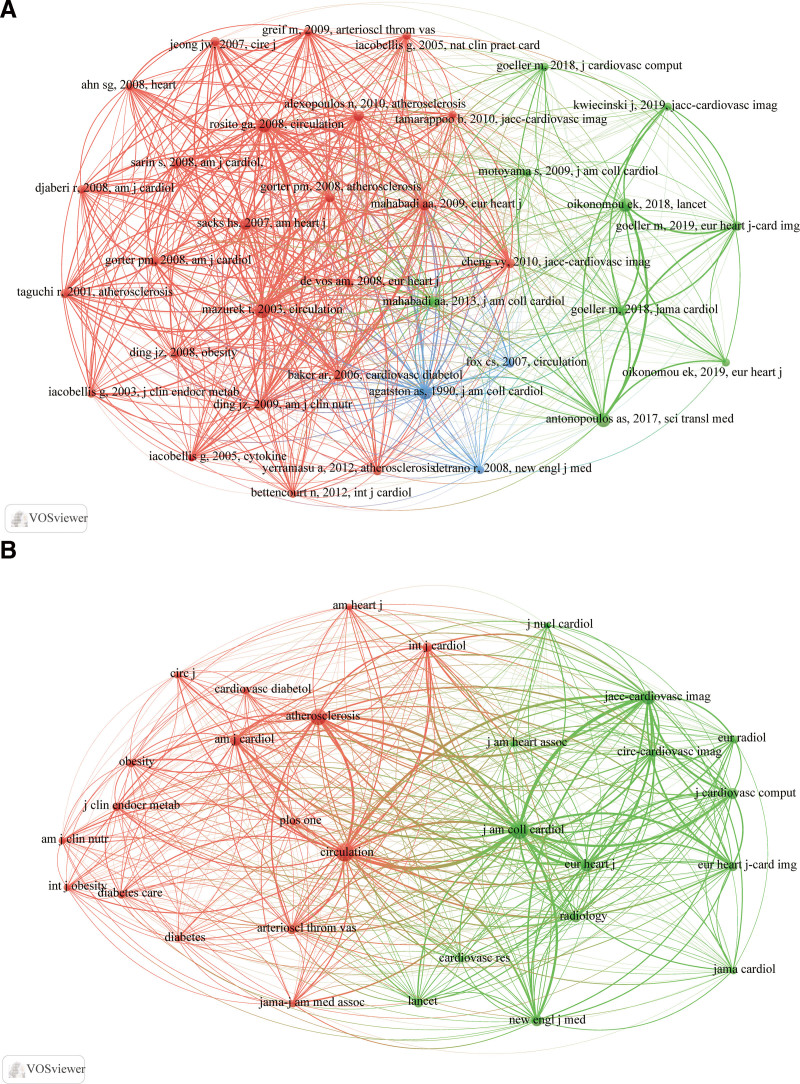
Co-citation network map of the cited references (A) and journals (B). The size of the nodes indicates the number of citations. The thickness of the lines represents the number of co-citations between 2 cited references (A) or journals (B).

Among the 11,679 cited references, a total of 36 cited references were analyzed with a minimum citation of 45 (Fig. 5A). The co-citation map displayed 3 distinct clusters in different colors. The red cluster accounted for the vast majority which also contained the most frequently cited reference. The time range of articles in red cluster was from 2001 to 2012. These studies gradually began to pay attention to the role of PAT and EAT in CAD. The green cluster primarily focused on articles related to PCAT, presenting its ability to improve the prediction and stratification of cardiac risk. Whereas the blue cluster consisting of 3 articles investigated the predictive value of coronary artery calcium in CAD. This may suggest that coronary calcification as the link between fat and CAD cannot be ignored.

To assess the distribution of cited references in the journal, we drew a co-citation network map of journals of cited references (Fig. 5B). Among the 1898 journals, we analyzed 30 journals with at least 170 citations, respectively. These journals were divided into 2 clusters, of which the red cluster was prone to be metabolism-related journals while the green cluster was mostly cardiovascular-related journals. Notably, The Journal of the American College of Cardiology stood out as the most frequently cited journal (1732).

### 3.4. Publication analysis of countries, institutions, and authors

**Figure 6.**
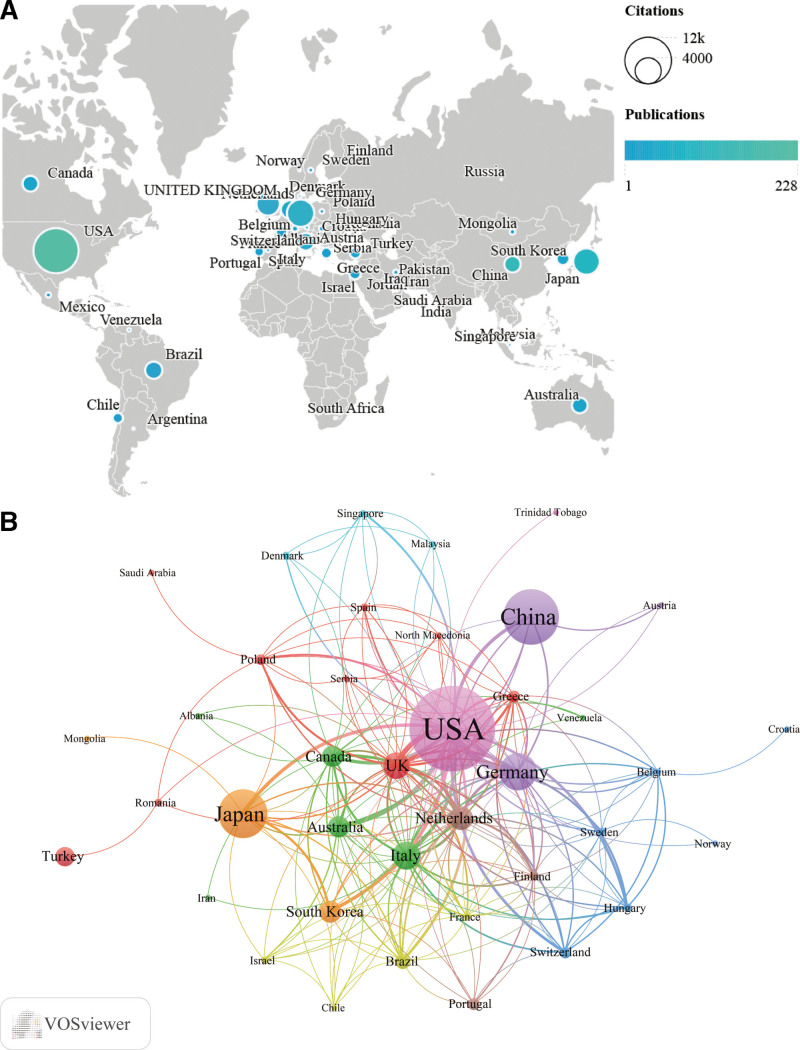
(A) Global distributions of publications and citations. (B) Collaboration network map of the co-authorship by countries. The size of the nodes indicates the number of publications. The thickness of the lines represents the strength of collaboration between them.

A total of 47 countries and 1002 institutions have participated in this field. We listed the top 10 countries which accounts for nearly 85% of all articles included (Table 2). The USA published the most number of articles (228), accounting for approximately 36% of the total dataset, which was followed by China (18%), and Japan (15%). In terms of citations, the USA continues to maintain its prominent place with up to 10,286 citations, followed by Germany (3818), and Japan (3542). The citations of Germany, the UK, the Netherlands, Canada, and Australia ranked higher than their publication counts (Fig. 6A). The research from these countries received attention and recognition within the scientific community irrespective of the relatively small publication counts. According to the H-index, the USA consistently held the first position (51), followed by Japan (30), Germany (29), and China (19). Figure 6B illustrates the collaboration networks of the co-authorship by 39 countries, because the rest 8 countries had no connections to other countries.

**Table 2:** Top 10 countries ranked by the number of publications.

Rank	Countries	Documents	Percentage	Citations	H-index
1	USA	228	36.25	10,286	51
2	China	118	18.76	1358	19
3	Japan	97	15.42	3542	30
4	Germany	64	10.17	3818	29
5	Italy	41	6.52	1573	17
6	UK	39	6.20	2807	19
7	Netherlands	33	5.25	1663	18
8	South Korea	29	4.61	821	16
9	Canada	27	4.29	1265	15
10	Australia	27	4.29	1250	15

In the list of top 10 institutions based on the publications counts shown in Table 3, there were 6 American institutions and 2 Japanese institutions, followed by the UK, and Australia with 1 institution. Cedars Sinai Medical Center took the lead as the most proactive institution with 54 publications accounting for 8.59% of the whole dataset. Wake Forest University got the highest average citations of 101.93. Table 4 presents a overview of the authors who had notable influence in their respective areas of expertise. Dey Damini made the most substantial research with 50 publications. The rank of top authors aligns with that of their respective affiliations. Antoniades Charalambos took the lead with 106.60 average citations.

**Table 3:** Top 10 institutions ranked by the number of publications.

Rank	Institutions	Documents	Percentage	Citations	Average citations
1	Cedars Sinai Medical Center (USA)	54	8.59	2235	41.39
2	University of Oxford (UK)	18	2.86	1707	94.83
3	Emory University (USA)	17	2.70	974	57.29
4	Monash University (Australia)	17	2.70	412	24.24
5	University of California San Diego (USA)	16	2.54	793	49.56
6	NIH National Heart Lung Blood Institute (USA)	15	2.38	1075	71.67
7	Wake Forest University (USA)	14	2.23	1427	101.93
8	Harvard Medical School (USA)	14	2.23	275	19.64
9	Tokyo Medical Dental University (Japan)	14	2.23	138	9.86
10	Tsuchiura Kyodo Hospital (Japan)	13	2.07	138	10.62

**Table 4:** Top 10 authors ranked by the number of publications.

Authors	Institutions	Documents	Citations	Average citations
Dey, Damini	Cedars Sinai Medical Center	50	2144	42.88
Achenbach, Stephan	University of Oxford	24	2146	89.42
Berman, Daniel S.	Emory University	24	1730	72.08
Slomka, Piotr J.	Monash University	23	1533	66.65
Gransar, Heidi	University of California San Diego	17	1271	74.76
Marwan, Mohamed	Harvard Medical School	16	1416	88.50
Lin, Andrew	NIH National Heart Lung Blood Institute	16	401	25.06
Antoniades, Charalambos	Wake Forest University	15	1599	106.60
Wong, Dennis T.	Tokyo Medical Dental University	14	409	29.21
Kakuta, Tsunekazu	Tsuchiura Kyodo Hospital	13	138	10.62

## 4. Discussion

A bibliometric analysis of literature published between 1992 and 2023 reveals a rapidly growing trend in adipose tissue and CAD research, indicating that this is a promising field with great potential. The field of adipose tissue research has grown rapidly in recent years, with increasing attention being paid to the anatomical proximity of adipose tissue to the coronary arteries. The general research trend revealed by the time overlay visualization analysis of keywords is consistent with that observed in the co-citation analysis of cited references and highly cited publications. It is mainly divided into 3 phases, with most research focusing on VAT, EAT, and PCAT, respectively. These are also the 3 keywords most frequently mentioned. Source co-citation analysis suggests that adipose tissue is associated with metabolism and cardiovascular health.

Studies in the first phase (1992–2007) began to break the boundaries of the traditional obesity indicator, BMI, which does not take into account individual variations in fat distribution. In the list of highly cited articles, the first article found that CAD patients with high VAT area were more likely to have multiple coronary risk factors such as hyperlipidemia, hyperglycemia, and hypertension, compared to controls with normal VAT area.^[14]^ Another study also highlighted the important role of VAT accumulation as a risk factor for atherosclerosis independent of BMI.^[17]^ As the only review in the list, the most cited study by Neeland et al^[8]^ highlighted that VAT can be accurately measured by CT and is an independent risk marker for cardiovascular and metabolic diseases. In addition, their proposal of “ectopic fat depots” may help to better understand the role of adipose tissue in the development of CAD, referring to fat that is not physiologically stored in the area of the body such as the liver, pancreas, heart, and skeletal muscle, which benefit from techniques with improved spatial resolution.^[8]^

Subsequent studies of the second phase (2008–2016) focused more on the adipose tissue surrounding the heart, including EAT (located between the myocardium and epicardium/visceral pericardium) and PAT (located outside the pericardial sac around the heart). EAT is mainly quantified in terms of its volume (EFV). As shown in Table 1, several studies consistently suggested that an increase in PAT and EAT was associated with CAD.^[10–13,15,16]^ EFV may provide additional assistance in identifying hemodynamically significant coronary stenosis.^[18,19]^ Similarly, only EFVi (EFV indexed to body surface area) emerged as a significant independent predictor of impaired myocardial flow reserve in a multivariate logistic regression analysis adjusting for age, sex, number of risk factors, and coronary artery calcium score (CACS).^[20]^ In addition, EAT was significantly associated with high-risk plaques, but not with CACS.^[21]^ The lack of the association between EAT and CAC was also shown in a study by Martins et al,^[22]^ in line with the finding in a meta-analysis that the association did not remain significant in the multivariable models.^[23]^ While another finding of a strong association of EAT with CAC progression,^[24]^ especially in young subjects and those with low CACS, suggested that EAT may promote early atherosclerosis development, showing that the association between EFV and CAD may represent different stages in the natural history of the disease. In fact, few studies have focused on PAT, as the term PAT was primarily used to refer to EAT, or included itself and EAT in most early research. The keyword analysis also showed that EAT appeared more frequently than other adipose tissue.

In the third phase (2017–2023), a notable paper by Antonopoulos et al^[2]^ shifted people’s focus to PCAT (a specific type of perivascular adipose tissue, directly around coronaries in a radial distance from the vascular wall equal to the luminal diameter of the adjacent vessel), which serves as a “thermometer” of adjacent vascular inflammation. They described a novel imaging biomarker (FAI) to track these changes in lipid content/size of adipocytes around coronary arteries. The fat attenuation index (FAI) was defined as the standardized average attenuation of PCAT after adjustment for technical, anatomical and biological factors. FAI was shown to be inversely related to adipocyte differentiation/size. In other words, the greater the adipocyte differentiation/size, the more lipophilic components in the tissue, resulting in more negative FAI values. Immediately the CRISP-CT study showed that a cutoff value of FAI ≥ ‐70.1HU can identify high-risk individuals with an approximately 3-fold risk of all-cause mortality and a 5-fold to 9-fold of cardiac mortality.^[9]^ Oikonomou et al used CCTA-based radiomic profiling of PCAT to propose a novel AI-powered imaging biomarker (fat radiomic profile, FRP) to describe fibrosis and vascularity, leading to a remarkable enhancement of cardiac risk prediction.^[25]^ Given the combination of radiomics and artificial intelligence, research on adipose tissue depots has flourished in recent years, accounting for more than a third of the total number of publications. Studies in the near future promise unexpected discoveries and a major breakthrough.

Indeed, PCAT, EAT, and PAT are all classified as forms of VAT. Adipocytes in VAT are characterized by smaller size, poorer differentiation and highly active secretion of adipokines, and their secretome is closely related to systemic metabolic status and cardiovascular risk.^[26]^ Considering its anatomical contiguity with the myocardium and coronary arteries without a clear boundary, EAT and PCAT may affect coronary arteries more directly. In some special groups such as diabetes mellitus, metabolic syndrome and HIV, adipose tissue may also serve as a valuable marker for identifying individuals at high risk for CAD.^[27–32]^ Moreover, quantification of adipose tissue by CCTA may be used as a useful tool for detecting drug efficacy in follow-up patients.^[33,34]^

This study had several limitations. Only WoSCC was used as the core data source, which may lead to potential biases in the analysis due to the uniformity and inaccuracy of the database. This bibliometric study typically relies on published literature and focus more on highly cited literatures. Studies with positive or statistically significant results are more likely to be published, while studies with null or negative findings may be less likely to be reported, leading to an incomplete representation of the research landscape.

## 5. Conclusion

Quantitative analysis of adipose tissue by CT, especially EAT and PCAT, was an important biomarker for predicting CAD risk and progression, the research on this topic experiencing a surge in popularity. The bibliometric analysis showcases the collaborative efforts among researchers, institutions, and countries worldwide in advancing this field of research, emphasizing the significance of understanding the connection between adipose tissue and CAD. However, for effective clinical translation, the primary issue that future research needs to address is the establishment of standardized measurement methods. Then large-scale clinical data are required to validate the utility of CT-based assessment of adipose tissue including evaluating its diagnostic accuracy, prognostic value, and potential impact on patient management and outcomes. Moreover, integration of multimodal imaging will help obtain a more comprehensive understanding of adipose tissue characteristics and their relationship with CAD.

## Author contributions

**Methodology:** Wenyue Shen, Kai Jin.

**Supervision:** Xiaojun Guan, Qijing Zhou, Xiaojun Xu.

**Visualization:** Jiayi Fu.

**Writing – original draft:** Jiayi Fu.

**Writing – review & editing:** Jiayi Fu, Rajiv Baichoo, Xing Xiong, Xiaojun Guan, Qijing Zhou, Xiaojun Xu.
